# *Bacillus*: A Biological Tool for Crop Improvement through Bio-Molecular Changes in Adverse Environments

**DOI:** 10.3389/fphys.2017.00667

**Published:** 2017-09-06

**Authors:** Ramalingam Radhakrishnan, Abeer Hashem, Elsayed F. Abd_Allah

**Affiliations:** ^1^Department of Biotechnology, Yeungnam University Gyeongsan, South Korea; ^2^Botany and Microbiology Department, College of Science, King Saud University Riyadh, Saudi Arabia; ^3^Mycology and Plant Disease Survey Department, Plant Pathology Research Institute Giza, Egypt; ^4^Plant Production Department, College of Food and Agricultural Sciences, King Saud University Riyadh, Saudi Arabia

**Keywords:** *Bacillus*, crop plants, diseases, heavy metals, drought, salinity

## Abstract

Crop productivity is affected by environmental and genetic factors. Microbes that are beneficial to plants are used to enhance the crop yield and are alternatives to chemical fertilizers and pesticides. *Pseudomonas* and *Bacillus* species are the predominant plant growth-promoting bacteria. The spore-forming ability of *Bacillus* is distinguished from that of *Pseudomonas*. Members of this genus also survive for a long time under unfavorable environmental conditions. *Bacillus* spp. secrete several metabolites that trigger plant growth and prevent pathogen infection. Limited studies have been conducted to understand the physiological changes that occur in crops in response to *Bacillus* spp. to provide protection against adverse environmental conditions. This review describes the current understanding of *Bacillus*-induced physiological changes in plants as an adaptation to abiotic and biotic stresses. During water scarcity, salinity and heavy metal accumulate in soil, *Bacillus* spp. produce exopolysaccharides and siderophores, which prevent the movement of toxic ions and adjust the ionic balance and water transport in plant tissues while controlling the pathogenic microbial population. In addition, the synthesis of indole-3-acetic acid, gibberellic acid and1-aminocyclopropane-1-carboxylate (ACC) deaminase by *Bacillus* regulates the intracellular phytohormone metabolism and increases plant stress tolerance. Cell-wall-degrading substances, such as chitosanase, protease, cellulase, glucanase, lipopeptides and hydrogen cyanide from *Bacillus* spp. damage the pathogenic bacteria, fungi, nematodes, viruses and pests to control their populations in plants and agricultural lands. The normal plant metabolism is affected by unfavorable environmental stimuli, which suppress crop growth and yield. Abiotic and biotic stress factors that have detrimental effects on crops are mitigated by *Bacillus*-induced physiological changes, including the regulation of water transport, nutrient up-take and the activation of the antioxidant and defense systems. *Bacillus* association stimulates plant immunity against stresses by altering stress-responsive genes, proteins, phytohormones and related metabolites. This review describes the beneficial effect of *Bacillus* spp. on crop plants, which improves plant productivity under unfavorable climatic conditions, and the current understanding of the mitigation mechanism of *Bacillus* spp. in stress-tolerant and/or stress-resistant plants.

## Introduction

The growth and yield of crop plants depend on genetic and variable environmental factors (Kleinwechter et al., 2016; Li et al., 2016). Plant breeding and genetic transformation approaches are used to transfer desired genes from crop varieties via sexual hybridization and artificial insertion, respectively, to develop new cultivars with the desired traits, such as high yield and adaptation to unfavorable environmental conditions (Jain, 1998). There is less commercial success for genetically modified agricultural crops. Numerous microbes are naturally beneficial to plants and help to sustain plant growth and yield during abiotic and biotic stresses. Plant cell chloroplasts and mitochondria evolved from symbiotic bacteria (Martin et al., 2001), and these key organelles absorb and convert energy for plant growth and survival. Plant-beneficial bacteria and fungi, living in the soil as free organisms or as endophytes, that trigger plant growth and protect plants from diseases and abiotic factors have been well documented by several researchers (Tonelli et al., 2010; Radhakrishnan et al., 2014). Some of the bacteria belonging to the *Acetobacter, Azospirillum, Azotobacter, Bacillus, Burkholderia, Klebsiella, Pseudomonas*, and *Serratia* genera have been recorded as plant growth-promoting bacteria (PGPB) (Glick, 1995; Jones et al., 2007). Among several species of PGPB, the *Pseudomonas* and *Bacillus* spp. have been identified as the predominant communities (Kang et al., 2015a), and a few of the PGPB have been commercialized due to their survival within a diverse range of biotic and abiotic environments. The first commercial bacterial fertilizer, Alinit, was developed from *Bacillus* spp. and resulted in a 40% increase in crop yield (Kilian et al., 2000). Other *Bacillus* spp.-based products, such as Kodiak (*Bacillus subtilis* GB03), Quantum-400 (*B. subtilis* GB03), Rhizovital (*Bacillus amyloliquefaciens* FZB42), Serenade (*B. subtilis* QST713), and YIB (*Bacillus* spp.), have been commercialized for improving crop production (Brannen and Kenney, 1997; Ngugi et al., 2005; Cawoy et al., 2011). Indeed, *Bacillus*-based bio-fertilizers are more active compared to *Pseudomonas*-based fertilizers due to the more effective metabolite production and spore-forming character of *Bacillus* spp., which enhances the viability of cells in commercially formulated products (Haas and Defago, 2005).

*Bacillus* spp. are gram positive, ubiquitous in nature and recovered from all niches in the environment. These species have also been used to prepare medicinal, industrial and agricultural products (Lyngwi and Joshi, 2014). Bio-fertilizers can be used as alternatives to chemical fertilizers and pesticides and can provide new insights into enhancing plant growth and yield in the face of diseases (Choudhary, 2011). The plant-beneficial *Bacillus* spp. associate with roots or rhizospheres and develop biofilms to increase plant growth (Beauregard et al., 2013). The application of *Bacillus*-based fertilizers to soil can enhance the plant-available forms of nutrients in rhizospheres, control disease-causing pathogenic microbial growth and induce pest defense systems (Garcia-Fraile et al., 2015; Kang et al., 2015b). This review is focused on the growth-promoting potential of *Bacillus* spp. in crop plants and the involvement of these bacteria in reprogramming plant physiological changes to achieve abiotic and biotic stress tolerance.

## *Bacillus* spp. metabolites promote plant growth

Seed germination and plant growth are significantly influenced by the nutrients available in the soil. Plants absorb phosphorus (P) and nitrogen (N) from the soil through root transporters, but the bioavailable forms of P and N are limited in rhizospheres (De-Willigen, 1986; Robinson, 2001; Bidondo et al., 2012). The beneficial effect of *Bacillus* spp. to crop improvement is given in Table 1. *Bacillus* spp. convert the complex form of essential nutrients, such as P and N, to a simple available form that is used during uptake by plant roots (Kang et al., 2015a; Kuan et al., 2016). Phosphate is involved in nucleic acid, phospholipid, and adenosine triphosphate (ATP) metabolism, among other metabolic pathways, in plant cells (Theodorou and Plaxton, 1993). The secretion of phosphatases and organic acids from *Bacillus* spp. acidifies the surrounding environment to facilitate the conversion of inorganic phosphate into free phosphate (Kang et al., 2014a, 2015a). Additionally, N is an important component of proteins, nucleic acids and other organic compounds in plants, and the available form of N in soil is limited, which slows plant growth in natural habitats (Barker et al., 1974; De-Willigen, 1986). Some of the *Bacillus* spp. release ammonia from nitrogenous organic matter (Hayat et al., 2010). Ding et al. (2005) reported that some of the *Bacillus* spp. have the *nifH* gene and produce nitrogenase (EC 1.18.6.1), which can fix atmospheric N_2_ and provide it to plants to enhance plant growth and yield by delaying senescence (Kuan et al., 2016).

**Table 1:** Bio-fertilizer effect of *Bacillus* spp. on crop plants.

***Bacillus* species**	**Plant growth promotion**	**References**
*B. insolitus; B. subtilis; B. methylotrophicus*	Increase the length and biomass of shoot, roots and leaves	Ashraf et al., 2004; Barnawal et al., 2013; Radhakrishnan and Lee, 2016
*B. megaterium; B. subtilis*	Enhance fruits and grains yield	Kilian et al., 2000; Dursun et al., 2010
*B. pumilus; B. megaterium*	Solubilize the P and fix the N in soil and increase their transport to roots	Kang et al., 2014a; Kuan et al., 2016
*B. subtilis; B. methylotrophicus*	Synthesis of plant growth hormones (IAA, GAs, cytokinins and spermidines) trigger plant growth	Arkhipova et al., 2005; Xie et al., 2014; Radhakrishnan and Lee, 2016
*B. subtilis; B. mojavensis*	Secretes ACC deaminase to inhibit plant senescence	Xu M. et al., 2014; Pourbabaee et al., 2016
*B. megaterium; B. methylotrophicus*	Enhance the endogenous proteins, amino acids, sugars, photosynthetic pigments and minerals (K, Mg, Na, P, Fe, Zn, and N) in plants	Kang et al., 2014a; Radhakrishnan and Lee, 2016

The iron-chelating properties of *Bacillus* spp. via siderophore production help to solubilize iron from minerals and organic compounds in rhizospheres (Nadeem et al., 2012). Siderophores bind Fe^3+^ in complex substances and reduce the Fe^3+^ to Fe^2+^, which then enters plants (Walker and Connolly, 2008).

The presence of tryptophan and other bacterial food source compounds induces the synthesis of indole-3-acetic acid (IAA) and other hormones in bacterial populations (Glick, 2014). Plant-growth-promoting substances, such as IAA, gibberellins, cytokinins and spermidines, are synthesized by *Bacillus* spp. and increase root and shoot cell division and elongation (Arkhipova et al., 2005; Xie et al., 2014; Radhakrishnan and Lee, 2016). The secretion of ACC deaminase (EC 4.1.99.4) by *Bacillus* spp. inhibits ethylene synthesis in crop plants and promotes plant growth (Xu M. et al., 2014; Pourbabaee et al., 2016). ACC deaminase breaks down ACC into ammonia and ketobutyrate in plant cells, and the cross-talk between ACC deaminase and IAA facilitates the reduction of ethylene, thereby enhancing plant growth (Honma and Shimomura, 1978; Glick, 2014). The N fixation, P solubilization, plant growth promoting hormones and enzymes section of *Bacillus* spp. confirm their bio-fertilizer effects on plants to improve the growth and yield of crops.

## *Bacillus*-mediated plant growth promotion under abiotic stress conditions

### Plant drought tolerance by *Bacillus* spp. inoculation

**Figure 1:**
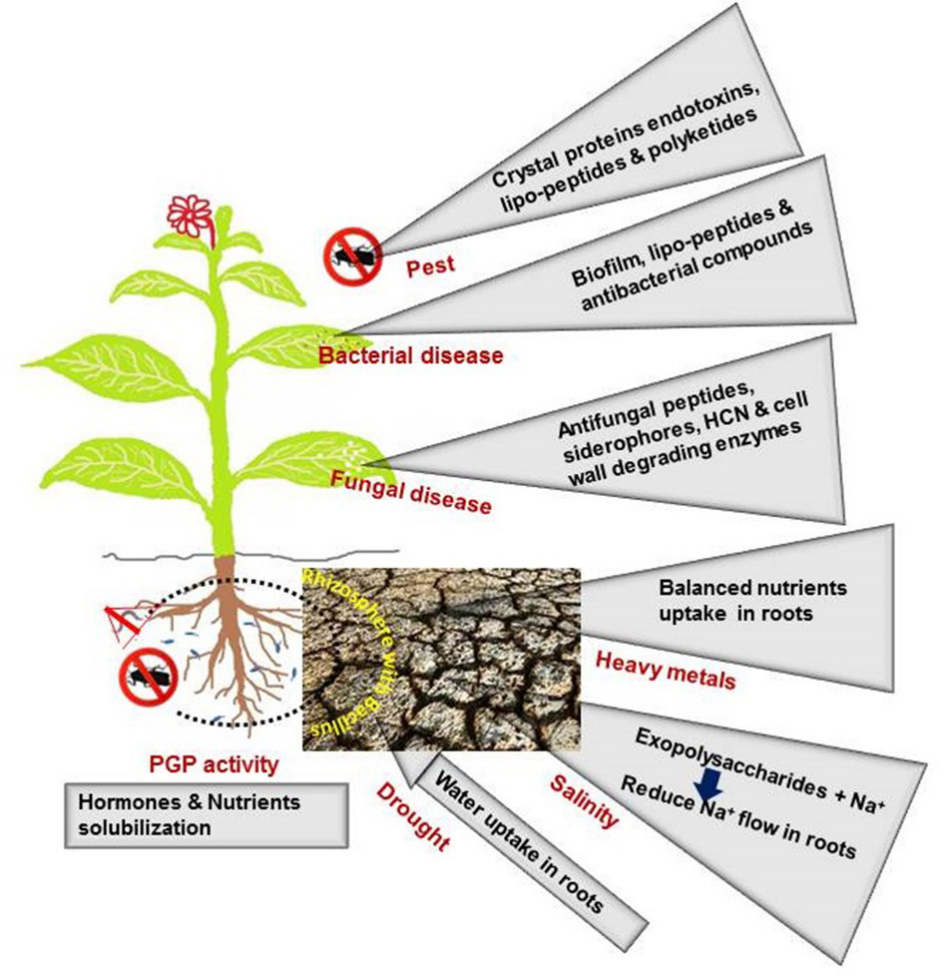
Direct effect of *Bacillus*-secretions on plant protection from adverse environments.

Soil moisture severely influences crop productivity in arid and semiarid areas. Low moisture content in the soil due to low annual precipitation creates drought stress in plants. Regulating the uptake and distribution of nutrients, transport of water, and accumulation of compatible solutes and antioxidants in plant tissues can help to improve plant productivity under drought conditions (Boomsma and Vyn, 2008). Applying drought-tolerant *Bacillus* spp. to the soil increases the populations of these bacteria on the roots and stimulates root exudation to promote both bacterial and plant growth (Sandhya et al., 2011). Plants colonized by *Bacillus* spp. take up more water, which is an important mechanism for plant protection against drought-induced damage (Marulanda et al., 2009). The mitigating effects of *Bacillus*-induced physiological changes in plants are shown in Figure 1 and Table 2.

**Table 2:** *Bacillus*-induced physiological and biochemical changes in crop plants during drought, salinity and heavy metal accumulation in soil and pest, pathogenic bacterial and fungal infection.

**Stress factors**	**Plant functions**		**References**
	**Stressed plants**	**Stressed plants with *Bacillus***	
**DROUGHT**			
	Reduce plant growth Decrease the water, nutrients (N, P, K, Ca, Mg, Zn, Cu, Mn, and Fe) and pigments Up or down regulate the antioxidants (CAT, SOD, POD, APX, and GR), hormones (SA, JA, and ABA) and drought responsible genes	Enhance plant growth Increase the water, nutrients (N, P, K, Ca, Mg, Zn, Cu, Mn, and Fe), pigments and hormones (SA, JA, and ABA). Up or down regulate the antioxidants (CAT, SOD, POD, APX, and GR) and drought responsible genes	Marulanda et al., 2009; Jumali et al., 2011; Barnawal et al., 2013; Castillo et al., 2013; Kasim et al., 2013; Armada et al., 2014, 2015; Timmusk et al., 2015; Kakar et al., 2016
**SOIL SALINITY**			
	Reduce plant growth Decrease the water, nutrients (N, P, K, Ca, Mg, S, Mn, Cu, and Fe), antioxidants (CAT and POD), pigments and hormones (IAA and GA). Increase the Na, Cl, ABA, and caspase activity; Up or down regulate the salt stress responsible genes	Enhance plant growth Increase the water, nutrients (N, P, K, Ca, Mg, S, Mn, Cu, and Fe), antioxidants (CAT and POD), pigments and hormones (IAA and GA). Decrease the Na, Cl, ABA and caspase activity; Up or down regulate the salt stress responsible genes	Ashraf et al., 2004; Jha and Subramanian, 2012; Mohamed and Gomaa, 2012; Karlidag, 2013; Nautiyal et al., 2013; Qurashi and Sabri, 2013; Kang et al., 2014b
**HEAVY METALS**			
	Reduce plant growth Decrease the water, nutrients (P, Ca, Fe, Mn, Zn, Cu, Cd Cr, and Pb) and pigments Up or down regulate the antioxidants (SOD, POD, APX, and DHAR)	Enhance plant growth Increase the water, nutrients (P, Ca, Fe, Mn, Zn, Cu, Cd Cr, and Pb) and pigments Up or down regulate the antioxidants (SOD, POD, APX, and DHAR)	Wani and Khan, 2010; Malekzadeh et al., 2012; Gururani et al., 2013; Wang et al., 2013; Jamil et al., 2014
**PEST**			
	Increase the larval population Reduce or stimulate the JA, ISR, and allelochemicals	Decrease the larval population Enhance the JA, ISR, and allelochemicals	Ben-Khedher et al., 2015a; Arrizubieta et al., 2016; Boukedi et al., 2016; Gadhave and Gange, 2016; Zebelo et al., 2016
**PATHOGENIC BACTERIA**			
	Increase the pathogenic bacterial population Decrease the defense enzymes (PAL, POD, PPO, SOD, CAT, and LOX), SA, pathogen resistant genes and proteins	Decrease the pathogenic bacterial population Increase the defense enzymes (PAL, POD, PPO, SOD, CAT, and LOX), SA, pathogen resistant genes and proteins	Chithrashree et al., 2011; Almoneafy et al., 2013; Kurabachew and Wydra, 2014; Jiang et al., 2015; Fousia et al., 2016; Hinarejos et al., 2016
**PATHOGENIC FUNGI**			
	Increase the pathogenic fungal population Up or down regulate the antioxidants (APX, GPX, POD, CAT, GR, PPO, and SOD), defense enzymes (PAL, chi, and glu), hormones (JA, ABA, IAA, GA, and SA)	Decrease the pathogenic fungal population Up or down regulate the antioxidants (APX, GPX, POD, CAT, GR, PPO, and SOD), defense enzymes (PAL, chi, and glu), hormones (JA, ABA, IAA, GA, and SA)	Liu et al., 2010; Chowdappa et al., 2013; Jain et al., 2013; Kang et al., 2015b; Kim et al., 2015; Narendra-Babu et al., 2015; Rahman et al., 2015; Yang et al., 2015

The uptake of N, P, and potassium (K^+^) decreases in drought-injured plants, whereas treatments with *Bacillus* spp. increase these macro nutrients in stressed plants (Barnawal et al., 2013). Bacterial enzymes increase the accumulation of the bioavailable forms of these macro nutrients in the soil and plants (Kang et al., 2015a; Kuan et al., 2016). In addition, these bacteria regulate high-affinity potassium transporter 1 (HKT1), which modulates Na^+^/K^+^ homeostasis, to mitigate drought stress (Gassmann et al., 1996; Vieira-Pires et al., 2013). Furthermore, K^+^ plays a key role in stomatal opening, turgor pressure maintenance, osmotic balance and controlling the transpiration rate in plants under drought stress (Loutfy et al., 2012). The scavenging activity of K^+^ helps to inhibit reactive oxygen species (ROS) formation during photosynthesis and NADPH oxidase metabolism (Cakmak, 2005). The concentrations of plant nutrients, such as Ca^++^, Mg^++^, Zn^++^, Mn^++^, and Cu^++^, are increased by *Bacillus megaterium, Bacillus thuringiensis* and *Bacillus* spp. applications in drought-stressed lavandula (*Lavandula angustifolia* L.) and salvia (*Salvia divinorum* L.) plants (Armada et al., 2014). Ca^++^ is involved in stabilizing membrane systems, and the accumulation of Mg^++^ regulates the homeostasis of ions in the chloroplasts, vacuoles and stomata of plant tissues (Shaul-Keinan et al., 2002; Huda et al., 2013). Some *Bacillus* spp., enhance plant growth during drought stress by increasing Fe levels. The high level of Fe^++^ in plants is probably due to siderophores derived from *Bacillus* spp. (Zawadzka et al., 2009). Aquaporins, particularly those encoded by the plasma membrane intrinsic protein (PIP) subfamily of aquaporin genes (Maurel et al., 2008), offer a low-resistance pathway for the movement of water across membranes to compensate for drought effects. *Bacillus* spp. regulate all the PIP genes to increase the hydraulic conductivity of roots in drought stressed plants, and aquaporins also transport urea, CO_2_ and H_2_O_2_ for N metabolism, carbon fixation and stress signaling, respectively (Armada et al., 2015).

The limited uptake of water and nutrients disturbs the normal cellular physiological processes and generates ROS, which damage proteins, lipids and nucleic acids in drought-exposed plants (Zgallai et al., 2005; Miller et al., 2010). ROS accumulation affects lipid membranes and causes lipid peroxidation and enhanced electrolyte leakage. Recovery from oxidative stress-induced damage is possible via antioxidant synthesis, which inhibits ROS formation. *Bacillus*-based bacterial association in plants can reduce the synthesis of ROS in cells via various scavenging enzymes (Kakar et al., 2016). *Bacillus* spp. either increase or decrease antioxidant enzyme activities in plants to mitigate drought stress. For example, *B. safensis* increases catalase (CAT; EC 1.11.1.6), superoxide dismutase (SOD; EC 1.15.1.1), peroxidase (POD; EC 1.11.1.7), ascorbate peroxidase (APX; EC 1.11.1.11) and glutathione reductase (GR; EC 1.6.4.2) activities (Chakraborty et al., 2013), while *B. amyloliquefaciens* decreases APX, GR and dehydroascorbate reductase (DHAR; 1.8.5.1) activities in plants under drought stress (Kasim et al., 2013).

The drought-induced oversynthesis of proline is suppressed in bacteria-treated plants, reflecting bacterial-derived resistance to the detrimental effects induced by drought (Barnawal et al., 2013). The normal metabolism of carbohydrates and amino acids is disturbed in plants during drought stress, and this effect is possibly reversed by interactions with *Bacillus* spp. Sucrose and fructose concentrations are enhanced in plants to contribute to drought adaptation by increasing root growth (Gagne-Bourque et al., 2016). As a consequence of stress, some of the endogenous amino acid accumulation triggers the production of secondary metabolites in stress-affected plants to mitigate oxidative stress (Jia et al., 2001). The synthesis of aromatic, glutamic and aspartic amino acid families is greater in plants associated with *Bacillus* spp. Histidine, tyrosine, phenylalanine, valine, leucine, isoleucine, asparagine, serine and γ-aminobutyric acid levels are increased due to the effects of drought in soil (Gagne-Bourque et al., 2016).

Drought inhibits pigment synthesis and reduces photosynthesis, while *Bacillus* spp. stimulate the synthesis of chlorophylls a and b and carotenoid in stressed plants, which increases photosynthesis (Barnawal et al., 2013; Hashem et al., 2015). The modulation of plant hormonal signals reprograms plant growth during drought stress. The hormone- and ACC deaminase-producing *Bacillus* spp. regulate plant growth by enhancing levels of stress-related hormones, such as salicylic acid (SA), jasmonic acid (JA) and abscisic acid (ABA), and reducing ACC, which is responsible for generating ethylene (Barnawal et al., 2013; Castillo et al., 2013). ABA accumulation improves drought tolerance by activating antioxidant enzymes and prevents water loss by stomatal closure (Lu et al., 2009; Zhu et al., 2011). Similarly, SA induces the expression of stress-related genes to maintain membrane stability and prevent the accumulation of ROS (El-Tayeb, 2005; Jumali et al., 2011).

The normal gene expression pattern in plants is altered during drought. The expression of *OsDIL* (drought-induced lipid transfer protein), *OsDREB1A* (dehydration-responsive element-binding protein 1A)*, OsGAPDH* (glyceraldehyde-3-phosphate dehydrogenase), *OsWRKY11* (WRKY transcription factor 11), *P4H* (prolyl-4-hydroxylase), *Cadhn* (dehydrin-like protein), *VA* (vacuolar H^+^-ATPase), *sHSP* (small heat shock protein), *CaPR-10* (pathogenesis-related protein 10), *cAPX* (cytosolic ascorbate peroxidase), *rbcL* (ribulose-1,5-bisphosphate carboxy/oxygenase large subunit) and *rbcS* (ribulose-1,5-bisphosphate carboxy/oxygenase small subunit) genes are affected during drought stress in crop plants. The lipid transfer is essential for development of lipidic orbicules and pollen exine formation, which is initiated by *OsDIL* genes (Zhang et al., 2010). *DREB* proteins involves in ABA-dependent and independent pathways to enhance the stress tolerance (Lata and Prasad, 2011). Similarly, *GAPDH* gene participates in cell proliferation, regulation of telomere length, apoptotic pathway and DNA repair (Kosova et al., 2017). *WRKY 11* gene induces systemic resistance through JA/ethylene mediated metabolic pathway (Jiang et al., 2016). However, *P4H* is responsible for hydroxylating proline-rich peptides influencing carbohydrate, lipid, protein and DNA metabolisms during plant growth and development (Asif et al., 2009). *DHN* genes confer stress tolerance due to the structural stabilization with chaperon-like activity to protect the macromolecules in cells (Koag et al., 2003; Porat et al., 2004). The pH is a fundamental factor for biological processes. Vacuolar H^+^-ATPase regulates the pH of cells, which results to stabilize the membrane, protein degradation, transport of small molecules and several metabolisms (Forgac, 2007). During heat and osmotic stresses, *sHSP* genes are expressed to conserve the metabolic reactions in cells (Schoffl et al., 1998). *PR-10* genes initiate and reprogramme the protein synthesis in pathogen infected or abiotic stress exposed plants (Xu P. et al., 2014). An antioxidant stimulating gene, *APX* plays a major role in redox condition of the electron transport machinery of chloroplast by regulating H_2_O_2_ levels (Davletova et al., 2005). In addition, *rbcL* and *rbcS* genes drive the synthesis of Rubisco enzymes for CO_2_ fixation during photosynthesis (Andersson and Backlund, 2008).

Some studies have revealed that tolerance against drought in bacteria-treated plants is associated with higher gene expression levels of *OsDIL, OsDREB1A, OsGAPDH, OsWRKY11, P4H, Cadhn, VA, sHSP, CaPR-10, cAPX, rbcL*, and *rbcS* gene expression (Khan et al., 2011; Wang et al., 2012; Kasim et al., 2013; Lim and Kim, 2013; Timmusk et al., 2015) and lower gene expression levels of *APX1* (ascorbate peroxidase 1), *SAMS1* (*S*-adenosyl-methionine synthetase 1, which acts as methyl donor and a precursor to polyamines synthesis (Sofia et al., 2001) and *HSP17.8* (heat shock protein 17.8) gene expression (Kakar et al., 2016). Plant drought tolerance may be accomplished by the interaction with *Bacillus* spp. resulting in enhanced water uptake, transport of nutrients, synthesis of hormones and pigments, and by the regulation of drought stress related genes and enzymes.

### *Bacillus* spp. applications to improve plant health in saline soil

Climatic changes in the environment affect regular rainfall each year. Salinity in agricultural land has been spreading worldwide due to low rainfall, high water evaporation rates and improper irrigation practices (Al-Karaki, 2006). The accumulation of salt in soil reduces the soil water potential and affects water and nutrient uptake by plant roots (Porcel et al., 2012). Under conditions of salinity, crop plants face disorder in several metabolic pathways, such as those related to photosynthesis, respiration, redox system homeostasis, phytohormone regulation, and carbohydrate and amino acid synthesis, which leads to reduced seed germination, plant growth and yield (Munns and Tester, 2008; Rady, 2011; Radhakrishnan and Lee, 2013, 2014). A microbial inoculation that includes *Bacillus* spp. can enhance plant growth during salt stress, which is an eco-friendly approach to sustainable agriculture (Radhakrishnan et al., 2014; Hashem et al., 2015, 2016a,b). The multiple plant growth-promoting characteristics (phosphate solubilization, ammonia, IAA and siderophore production) of *Bacillus licheniformis* A2 mitigate the detrimental effects of salt stress and increase plant growth in stressed peanut plants (Goswami et al., 2014). The association of *Bacillus* spp. with plants alters the plant metabolism in stressed plants to increase plant growth (Table 2). To tolerate salt stress, plants must prevent the excess uptake of Na^+^ and Cl^−^, but they need to continue the uptake of essential nutrients, such as K^+^ and ${\text{NO}}_{3}^{-}$ (Jeschke and Wolf, 1988). Exopolysaccharide (EPS) in the rhizosphere soil binds Na^+^ and inhibits Na^+^ transport into plant root cells (Figure 1). Inoculating wheat seedlings with EPS-producing *Bacillus insolitus* MAS17 and certain other *Bacillus* spp. covers the root zones with soil sheaths and restricts the passive flow of Na^+^ into the stele to mitigate salt stress effects (Ashraf et al., 2004). The bacteria-induced enhancement of the K^+^/Na^+^ ratio in plants grown in saline soil mitigates the effects of salinity stress (Han et al., 2014). The elevated levels of N, P, K, Ca, Mg, S, Mn, Cu, and Fe produced in salt-affected plants by the interaction with *Bacillus* spp. maintains plant growth during stress (Jha and Subramanian, 2012; Karlidag, 2013). The salt-tolerant bacteria increase the relative water content as well as the osmotic and turgor potential to improve the growth of salt-injured plants (Yang et al., 2016).

*Bacillus* spp. reduce the toxic effects of salinity in plants by inhibiting lipid peroxidation (Han et al., 2014). Hashem et al. (2015) proved that *B. subtilis* improved lipid synthesis, specifically that of oleic, linoleic, and linolenic acids as well as phospholipids, in plants grown under salt stress. This increase in lipid synthesis might mitigate lipid peroxidation and oxidative stress in the plants. ROS production is controlled by antioxidant enzymes. In bacteria-treated plants, APX and SOD activities are decreased, while nitrate reductase (NR; EC 1.7.1.1), CAT and POD activities are enhanced (Jha and Subramanian, 2012, 2015). *Bacillus pumilus* associated with the roots of salt-stressed rice plants reduces the activity of caspase (Jha and Subramanian, 2014), which is a protease that belongs to the cysteine endopeptidase family and is involved in programmed cell death in plants (Groten et al., 2006). The reduction of caspase activity decreases ROS formation and programmed cell death and reprograms the action of antioxidants to accomplish plant tolerance (Jha and Subramanian, 2014). Salt-tolerant *B. subtilis* RH-4 improves seed germination and plant growth by enhancing the synthesis of photosynthetic pigments, carbohydrates, proteins and osmolytes, such as proline, glycine betaine and choline, in salt-injured chickpea plants (Qurashi and Sabri, 2013). The regulation of these primary metabolic pathways in plants to protect against salinity-induced disorders promotes crop tolerance. In addition, some of the secondary metabolites, such as gallic acid, caffeic acid, syringic acid, vanillic acid, ferulic acid, cinnamic acid, and quercetin, are increased in plants associated with bacteria, which allows plants to tolerate salt stress (Tiwari et al., 2011).

The regulation of hormones under stress conditions is a complex phenomenon. Hormone levels are up- or down-regulated by environmental factors. ABA accumulation in plants grown under salt stress induces stomatal closure to reduce water loss and increases salt tolerance via stress responsive genes (Leung and Giraudat, 1998; Herrera-Medina et al., 2007), while bacterial inoculation decreases the stress-induced ABA synthesis and protects plants from the effects of stress (Kang et al., 2014b). Similarly, *Bacillus* spp. can produce plant hormones to enhance the concentrations of IAA and GA but reduce the synthesis of ABA in plants grown under salt stress (Mohamed and Gomaa, 2012). Several gene families are affected by salinity, and their transcriptional disorder retards plant growth. However, bacteria stimulate the expression of the *NADP-Me2* (NADP malic enzyme 2), *EREBP* (ethylene-responsive element-binding protein), *SOSI* (salt overly sensitive 1), *BADH* (betaine aldehyde dehydrogenase) and *SERK1* (somatic embryogenesis receptor-like kinase 1) genes, while the *GIG* (gigantea) and *SAPK4* (serine threonine protein kinase) genes in plants are down-regulated due to salinity (Nautiyal et al., 2013). Overall reports suggest that plants can tolerate soil salinity by the effect of *Bacillus* spp. induced regulation of several genes, proteins, antioxidant enzymes, pigments, hormones, nutrient transport and prevention of excess sodium transport in plant system.

### The influence of *Bacillus* spp. on plant growth during heavy metal accumulation in soil

Agricultural lands contaminated with trace metals deposited from industrial effluents and agro-chemicals affect the ecological food chain, including crop cultivation, and alter microbial communities (Hu et al., 2009; Ashraf et al., 2017). Accumulated Cu, Mn, and Zn are considered major pollutants in soil and water, and these metals cannot be easily degraded into harmless substances (Ma et al., 2009; Arthur et al., 2012). Chelators are used to reduce metal toxicity but are also harmful to living organisms (Tandy et al., 2006). In contrast, microorganisms solubilize or convert toxic metals to non-toxic forms, which is applicable to the integrated management of heavy metal phytoremediation (Bosecker, 1997; Kang et al., 2015c). The inoculation of *Bacillus* spp. into heavy metal-contaminated soil can possibly reduce the toxic effects of these metals on plant growth. The bacteria support plant growth by increasing water uptake and reducing electrolyte leakage to mitigate Cd stress (Ahmad et al., 2014). *B*. *licheniformis* enhances Cu, Zn, Cd, Cr and Pb accumulation and distribution in plants grown in heavy metal-contaminated soil, which leads to reduced levels of toxic metals in soil (Brunetti et al., 2012). Similarly, excess amounts of Cd in soil reduce nutrient (P, Fe, Zn, and Mn) uptake in plants, but bacteria promote an increase in the levels of these nutrients in plants to protect against the effect of Cd (Malekzadeh et al., 2012 and Figure 1). The association of some *Bacillus* spp. increases the P and Ca contents and reduces Ni accumulation in plants grown in contaminated soil (Jamil et al., 2014). The beneficial effects of the interaction with *Bacillus* spp. (enhancement of water, ions, pigments and enzymes) in heavy metal-affected plants are shown in Table 2.

Heavy metal deposition in soil affects the redox state of plant metabolism by inducing signaling molecules such as ROS. Excess ROS generation damages lipid membranes and causes lipid peroxidation (Kang et al., 2015c). The contamination of soil with the most common industrial toxic metals, Pb and As, accelerates lipid peroxidation in affected plants. *Bacillus* spp. alleviate this stress effect by reducing lipid peroxidation and SOD activity and increasing amylase and protease to promote plant growth in heavy metal-polluted soil (Pandey et al., 2013). Similarly, bacteria support plant tolerance against Zn and Cu stress by enhancing the activities of ROS scavenging enzymes, such as POD, SOD, CAT, APX, and DHAR (Gururani et al., 2013; Wang et al., 2013). Cr stress reduces acid phosphatase activity in plants, but bacterial treatment elevates the activity of this enzyme (Riaz et al., 2010). The regulation of antioxidants in cells inhibits oxidative stress damage and triggers plant growth-promoting substances to enable plants to adapt to metal stress. *Bacillus*-mediated plant tolerance against Ni and Cr stresses is achieved through the enhancement of photosynthetic pigments and leghemoglobin, which leads to increased crop yield (Wani and Khan, 2010; Jamil et al., 2014). The plant growth and survival at heavy metal polluted soil can be achieved by increasing the balanced uptake of mineral nutrients and pigments synthesis, and also modulating the endogenous antioxidants due to the association of *Bacillus* spp. To understand the mitigation mechanisms of *Bacillus* spp. against heavy metal pollution, more plant physiological studies are required.

## Mitigation of biotic stresses in plants by *Bacillus* spp. inoculation

### *Bacillus*-induced pest control and plant protection

In organic farming, the use of bacterial agents is considered an environmentally friendly and safe method to increase crop productivity in the presence of pests (Dihazi et al., 2012). Plant-beneficial *Bacillus* spp. reduce the use of chemical fertilizers and pesticides for the sustainable production of various crops in modern agriculture (Myresiotis et al., 2015). For example, thiamethoxam is an insecticide used to control an extensive range of pests, such as aphids, beetles, lepidopteran species, thrips and whiteflies (Karmakar and Kulshrestha, 2009), but this compound causes a decline in insects beneficial to plants, such as honey bees; therefore, the use of this chemical in seed coating has been banned by the European Union (Girolami et al., 2009). Alternatively, eco-friendly microbial pesticides can fill the gap formed by the discontinuation of chemical pesticides use in the field of agriculture. A well-known bio-insecticide, *B. thuringiensis*, can control a broad range of diverse insects for pest management in the agricultural field (Navon, 2000). For example, the insects *Helicoverpa armigera, Spodoptera littoralis, Oryzophagus oryzae, Spodoptera frugiperda*, and *Chilo partellus* are damaging to plant growth and fruit, but *B. thuringiensis* inhibits the larval growth of insects and increases plant growth and yield (Brownbridge, 2001; Berlitz et al., 2012; Benfarhat-Touzri et al., 2014; Arrizubieta et al., 2016) without affecting other microbial populations within the phyllosphere (Wang et al., 2014). Some other *Bacillus* spp., such as *B. cereus, B. subtilis*, and *B. amyloliquefaciens*, are also involved in pest control (Gadhave and Gange, 2016).

The mechanism of *Bacillus*-induced pest control in plants varies with pest species as well as plant genotype (Navon, 2000; Paramasiva et al., 2014; Mnif and Ghribi, 2015; Wielkopolan and Obrepalska-Steplowska, 2016). *Bacillus* spp. kill pest larvae and induce systemic resistance in plants (Table 2). Pesticide-producing *Bacillus* spp. in soil and roots support plant growth and increase the uptake and systemic translocation of pesticide (thiamethoxam) throughout the entire plant to control pest infestations (Myresiotis et al., 2015). *Bacillus* spp. colonize plant parts, including the phyllosphere, and larvae and/or adult pests ingest the *Bacillus*-containing plant tissues during feeding. A primary site of bacterial infection begins with extensive damage to the larval midgut epithelium by bacterial crystal proteins, which interact with chitin and peritrophic membranes (Vachon et al., 2012; Feng et al., 2015; Figure 1). During later stages of infection, *Bacillus* spp. crystal protein endotoxin, lipopeptides and polyketides (iturin, fengycin, surfactin, bacillomycin, bacillaene, macrolactin, and difficidin) modify the vacuolization of the cytoplasm, induce vesicle formation, lyse brush border membrane, and degenerate apical membranes, leading to damage of microvilli and finally causing larval death (Ben-Khedher et al., 2015a; Boukedi et al., 2016). Surfactin attaches to the Ca^2+^ receptor site and changes the peptide composition in the cellular phospholipid bilayer (Maget-Dana and Ptak, 1995), while iturin increases cell membrane permeability via the formation of ion-conducting pores (Maget-Dana and Peypoux, 1994). *Bacillus* spp. elicit the JA-pathway-related genes and simultaneously increase the gene expression for other secondary metabolites (allelochemicals, which inhibit pest larval growth) in plants to defend against pests (Zebelo et al., 2016). The obtained reports suggest that *Bacillus* spp. control the larval population of pest and trigger the ISR mechanism and allelochemicals in plants to prevent the pest damage.

### Bacterial disease prevention in plants by the application of *Bacillus* spp.

Plant disease-causing pathogenic bacteria, fungi, viruses and nematodes are major challenges in maintaining plant health and yield in agricultural lands (Hussey and McGuire, 1987; Guo et al., 2013; Narasimhan and Shivakumar, 2015). The application of plant-beneficial microorganisms is an alternative to chemical fungicides, bactericides and nematicides and an effective environmentally friendly approach to improving plant growth and controlling many plant diseases (Choudhary and Johri, 2009; Radhakrishnan et al., 2013; Adam et al., 2014; Egamberdieva et al., 2014). *Bacillus* spp. inhibit pathogenic microbial growth in soil and/or in plant tissues as well as the detrimental effects of the pathogens in plants. For example, pathogenic bacteria such as *Ralstonia solanacearum, Pseudomonas savastanoi* and *Xanthomonas axonopodis* infect plants and generate diseases, whereas *Bacillus* spp. inoculation suppresses pathogen growth and protects plants from diseases (Krid et al., 2012; Yi et al., 2013). Biofilm formation around the root surface by *Bacillus* spp. and their secretion of toxins (surfactin, iturin, macrolactin, bacillomycin, and fengycin) destroy the pathogenic bacterial populations and reduce disease incidence in plants (Chen et al., 2013; Huang et al., 2014; Elshakh et al., 2016; Hinarejos et al., 2016; Figure 1). The secretions of *Bacillus* spp. degrade the pathogenic bacterial cell walls and change the cell morphology to kill the pathogen (Elshakh et al., 2016).

In addition, pathogenic bacteria, including *R. solanacearum* and *Xanthomonas oryzae*, affect plant defense systems by decreasing phenylalanine ammonia-lyase (PAL; EC 4.3.1.24), POD, PPO, SOD, CAT, and lipoxygenase (LOX; EC 1.13.11) activities, but these defense enzyme activities are accelerated in diseased plants following the administration of *Bacillus* spp. (Chithrashree et al., 2011; Almoneafy et al., 2013; Kurabachew and Wydra, 2014; Table 2). PAL is involved in the biosynthesis of polyphenol compounds (lignin, flavonoids and phenylpropanoids) and triggering the plant resistance against environmental stimuli (Fritz et al., 1976; Tanaka et al., 1989). However, the systemic resistance to diseases induced in plants by *Bacillus* spp. is made possible by increasing SA content and the gene and protein expression of proteinase inhibitor II (*Pin2*) and pathogen resistant 1 (*PR1*) (Jiang et al., 2015; Fousia et al., 2016; Hinarejos et al., 2016).

### Effects of *Bacillus* spp. inoculation on crop protection from pathogenic fungi

The antagonistic activity of *Bacillus* spp. controls the mycelial growth of fungi, preventing plant fungal disease (Abdalla, 2015; Chowdhury et al., 2015a; Akram et al., 2016; Aydi-Ben-Abdallah et al., 2016) and increasing plant growth and yield (Narasimhan and Shivakumar, 2015). Populations of *Bacillus* spp. can be successfully established in the soil and root rhizospheres without any lasting effects on other bacterial populations (Chowdhury et al., 2015a). *Bacillus* spp. attach to the mycelial cell walls, and the chitosanase (EC 3.2.1.123), protease (EC 3.4.21.112), cellulase (EC 3.2.1.4), glucanase (EC 3.2.1.21), siderophores, and HCN of the bacteria crack and deform the hyphae, which leads to altered cell structure and functions due to vacuolation and protoplast leakage (Ben-Khedher et al., 2015b; Han et al., 2015; Narendra-Babu et al., 2015). Bacterially synthesized antifungal peptides, such as iturin, fengycin, mixirin, pumilacidin, surfactin, and a novel cyclic peptide with a molecular weight of 852.4 Da, are involved in the destruction of the pathogenic fungi in rhizospheres (Han et al., 2015; Yamamoto et al., 2015; Figure 1). *Bacillus* spp. mitigate pathogen-induced biotic stress via physiological changes (Table 2) in the photosynthetic and respiratory pathways and the regulation of carbohydrate, phenyl-propanoid and N metabolism and defense-related proteins in diseased plants (Jain et al., 2015). Gene expression patterns in plants are also altered during infection by pathogenic fungi, and a number of dependent genes are activated to protect the plant from biotic stresses. The expression of genes encoding β-1,3-glucanase (*PR-2*), chitinase (*PR-3* and *PR-4*), peroxidase (*PR-9*), lipid transfer protein (*PR-14*), metallothionein-like protein (*LfMT1*), oxalate oxidase (*LpOXO4*), lipoxygenase (*LOX*), and a putative defensin (*LpTHb*) are upregulated, whereas the putative glycine-rich protein (*LfGRP1*) and PsbR protein of photosystem 2 (*LfPsbR*) genes are downregulated in diseased plants treated with *Bacillus* spp. (Liu et al., 2010; Kim et al., 2015; Rahman et al., 2015). The gene expression of major antioxidants and defense enzymes, such as POD, PAL, SOD, CAT, and PPO, is also stimulated during *Bacillus* spp. treatment (Narendra-Babu et al., 2015; Yang et al., 2015). The higher levels of energy, metabolism and defense-related proteins in *Bacillus*-treated diseased plants induce systemic resistance (Sarosh et al., 2009). The stress-induced transcriptional changes in plants trigger the production of secondary metabolites and defense enzymes to reduce oxidative damage. Antagonistic *Bacillus* spp. reduce lipid peroxidation and increase antioxidant enzymes, such as APX, CAT, GR, GPX, POD, PPO, other defense enzymes, such as PAL, chitinase (EC 3.2.1.14), and β-1,3-glucanase (EC 3.2.1.39), and phenolic acids to alleviate the adverse effects of pathogenic infection (Solanki et al., 2012; Chowdappa et al., 2013; Jain et al., 2013). The activity of hormones in plant immunity is well documented, and in particular, the synthesis of SA and JA plays a major role in plant defense. The cross talk among hormones is a complex process that induces disease resistance. Chowdappa et al. (2013) and Kang et al. (2015b) proved that the plant growth-promoting hormones IAA and GA are increased in *Bacillus*-treated plants along with SA, while JA and ABA are decreased in pathogen-infected plants. However, the studies on beneficial effect of *Bacillus* spp. in plants against fungal diseases conclude that the detrimental effects due to fungal infection in plants can be mitigated during the inoculation of *Bacillus* spp. by reprogramming the activity of plant defense enzymes and hormones. Additional physiological and molecular studies are required to elucidate the bio-control mechanisms of *Bacillus* spp. against pathogenic fungi-induced disease in crops.

### *Bacillus* spp.-plant interactions for viral and nematode disease resistance

The second largest group of plant diseases after fungi is caused by viruses. The most effective method of virus control has been accomplished by chemical treatments. The prolonged use of chemicals leads to deposits in soil and increases the drug resistance of plant pathogens (Zhao et al., 2017). Some of the *Bacillus* spp., produce the antiviral compounds against pathogen (Esawy et al., 2011). Very few studies have reported on the bio-control effects of bacteria in preventing or resisting viral disease. The disease rate is reduced as a consequence of induced systemic resistance (ISR) by interaction with *Bacillus* spp., leading to enhanced plant growth during cucumber mosaic virus infection (Zhang et al., 2004). The biofilm formation and surfactin production from *B. amyloliquefaciens plantarum* defense the viral disease in plants by triggering ISR machinery (Chowdhury et al., 2015b). Similarly, *Bacillus* spp. induce systemic resistance against viral disease caused by tobacco mosaic virus by inhibiting viral coat protein synthesis and by increasing the expression of disease-resistant signaling genes (*Coil* and *NPR1*), defense genes (*PR-1a* and *PR-1b*) and cell wall expansin (*NtEXP2* and *NtEXP6*) genes in plants (Wang, 2009). The *NPRI* and *Coil* genes regulate the ISR- and JA-dependent pathways, respectively (Xie et al., 1998; Mou et al., 2003), which indicates that *Bacillus* spp. application can prevent viral damage in plants. However, crops are also damaged by nematodes, which are plant parasites and are recognized as a severe threat to plant growth. Root-knot nematodes have been recorded as the most damaging parasite relative to other types of nematodes worldwide. The host range of this nematode covers nearly 5500 plant species (Trudgill and Blok, 2001). The application of a bacterial inoculation controls the nematode populations. For example, *Bacillus* spp. prevent root-knot nematode infection in crops and develop resistance by reducing gall and egg masses in plants (Adam et al., 2014). Antimicrobial peptides, bacteriocins synthesized from *Bacillus* spp. inhibit the growth of pathogenic nematodes (Chowdhury et al., 2015b). Liu et al. (2013) identified the *PZN* gene cluster in *B. amyloliquefaciens*, and revealed that these genes are responsible for nematicidal activity against nematodes. In addition, the secretion of crystal proteins (Cry5B and Cry6A) from *Bacillus* spp. controls the growth of free-living (*Caenorhabditis elegans*) and plant-parasitic (*Meloidogyne hapla*) nematodes (Yu et al., 2015). Cry5B binds with glycolipids receptors, leading to intestinal damage in *C. elegans*. Moreover, Cry6A restricts the growth of nematodes by inhibiting egg hatch, motility and infection to host tissues (Kho et al., 2011; Yu et al., 2015). The documented results of *Bacillus* spp. against virus and nematode suggest that some of the metabolites synthesized from *Bacillus* spp. inhibit the viral and nematodes population and increase the plant resistance through the expression of defense genes.

## Conclusions

Crop productivity is decreasing due to climatic changes, and human populations are increasing daily, which results in starvation problems in under-developed countries. Research is ongoing to enhance crop yields despite various unfavorable environmental conditions. Physical, chemical and biological methods are being used to address the biotic and abiotic stress-induced damage in plants. The mutualistic relationship between plants and microbes is well known, especially the interactions between plants and bacteria either from the soil or inside the plants that help to improve the plant health under adverse stress conditions. The plant-beneficial *Bacillus* spp. produce plant growth-promoting substances (hormones and solubilizing enzymes) to increase plant growth. During drought and with salinity and heavy metal accumulation in the soil as well as pathogen infection, crop productivity is reduced, but the association with *Bacillus* ssp. promotes crop yield via various metabolites. Some of the physiological alterations in plants during *Bacillus* spp. inoculation in stress environments slow plant aging. For example, the ethylene-suppressing enzyme (ACC deaminase) synthesized by *Bacillus* spp. mitigates the detrimental effects of abiotic and biotic stress in plants by delaying senescence. Exopolysaccharide production by *Bacillus* spp. has been frequently reported to reduce sodium ion transport and regulate plant nutrient uptake during salinity stress. Additionally, the lipopeptides and toxic substances secreted from *Bacillus* spp. prevent pathogen growth and reduce disease occurrence in crops. The plant growth-promoting activities of *Bacillus* spp. have been well-documented as evidenced by increased growth of roots, shoots, and leaves as well as enhanced yields. However, very few studies have been conducted regarding the physiological and molecular aspects of these processes. Some of these studies have revealed that *Bacillus* spp. regulate nutrient uptake, water transport, and antioxidant, pigment, hormone and stress-responsive genes and proteins in plants leading to tolerance under adverse environmental conditions. This review concludes that *Bacillus* spp. are biological organisms that can potentially induce stress tolerance in plants, and more genomics, proteomics and metabolomics studies are required to elucidate the mechanism of *Bacillus*-plant interactions for biotic and abiotic stress management in crops.

## Author contributions

RR, AH and EA collected the research article information and wrote and revised the article together in a parallel manner. All the authors approved the final version of this manuscript.

### Conflict of interest statement

The authors declare that the research was conducted in the absence of any commercial or financial relationships that could be construed as a potential conflict of interest.
